# Comparative Evaluation of Microbial Growth on Unfilled and Filled Polyetheretherketone (PEEK) With and Without Surface Treatments: An In Vitro Study

**DOI:** 10.7759/cureus.92227

**Published:** 2025-09-13

**Authors:** Sowmya Kumar, Vidhyasankari N, Mathew Chalakuzhiyil Abraham, Maheshwaran Marappan, Biju K A, Vishnupriya Venkatasubramanian, Aishwarya A Nair

**Affiliations:** 1 Department of Prosthodontics and Crown and Bridge, KSR Institute of Dental Science and Research, Tiruchengode, IND

**Keywords:** antimicrobial properties, bacterial adhesion, biofilm formation, laser treatment, peek, plasma treatment, surface modification

## Abstract

Aims and background: Polyetheretherketone (PEEK) is a high-performance thermoplastic polymer used in biomedical applications due to its biocompatibility, mechanical strength, and chemical resistance. However, its inherent hydrophobicity and bio-inertness promote microbial adhesion and biofilm formation, limiting its effectiveness. This study evaluates the effects of laser and plasma surface treatments on the surface characteristics and microbial adhesion of PEEK.

Materials and methods: PEEK samples, both unfilled and glass-filled, were subjected to laser (neodymium-doped yttrium aluminum garnet (Nd:YAG), 1064 nm) and argon plasma treatments. Surface morphology was analysed using scanning electron microscopy (SEM). Bacterial adhesion and biofilm formation were evaluated using crystal violet staining and spectrophotometric analysis at 570 nm. Live/dead staining was performed to determine bacterial viability.

Results: Laser surface treatment produced uniform microstructures and significantly reduced bacterial adhesion and biofilm formation across all tested strains (*E. coli, S. mutans, and S. oralis*). Plasma treatment enhanced the surface wettability and reduced the microbial adhesion, to a lesser extent compared to laser surface treatment. Filled PEEK demonstrated higher roughness but lower microbial growth due to the antimicrobial effects of embedded glass fibers. Statistical analysis confirmed that biofilm formation was significantly lower in surface-treated groups (p < 0.001), exhibiting the antimicrobial performance.

Conclusion: Laser surface treatment with an Nd:YAG laser was the most effective in reducing bacterial viability and biofilm formation, making it a promising technique for biomedical applications where infection control is essential. Plasma treatment remains a viable alternative for surface functionalization and biocompatibility enhancement. The findings emphasise the importance of surface modifications in improving the antimicrobial properties of PEEK for medical applications.

Clinical significance: Surface modifications of PEEK via laser and plasma treatments provide promising strategies for reducing bacterial adhesion and biofilm formation, improving the material’s suitability for biomedical implants and prosthetic applications.

## Introduction

Polyetheretherketone (PEEK) is a high-performance thermoplastic polymer widely used in medical, electronic, and aerospace applications due to its chemical resistance, mechanical strength, and biocompatibility [1]. In dentistry, it serves as an alternative to metals and ceramics in implants, prosthetic frameworks, and orthodontic components, offering advantages such as reduced metal allergies, improved aesthetics, and precise milling [2]. Despite its benefits, PEEK's hydrophobicity and bio-inertness can limit its effectiveness in biomedical applications by promoting microbial adhesion and biofilm formation [3]. To address this, surface modification techniques like laser and plasma treatments are employed. Laser treatment modifies PEEK’s surface by creating micro- and nanoscale roughness, reducing bacterial colonisation while enhancing cell adhesion [4]. Neodymium-doped yttrium aluminum garnet (Nd:YAG) laser treatment has shown improvements in wettability and bioactivity, like effects observed in other polymers. Plasma treatment, particularly with ionised argon gas, alters surface chemistry by breaking covalent bonds and removing contaminants, enhancing roughness and hydrophilicity [5,6]. These modifications improve cell attachment while reducing bacterial adhesion [7,8]. Argon plasma treatment is especially effective due to its controlled modification process without introducing unwanted chemical changes [9]. This study evaluates the effects of laser and plasma surface treatments on PEEK’s surface characteristics and microbial adhesion.

## Materials and methods

Study design

**Figure 1:**
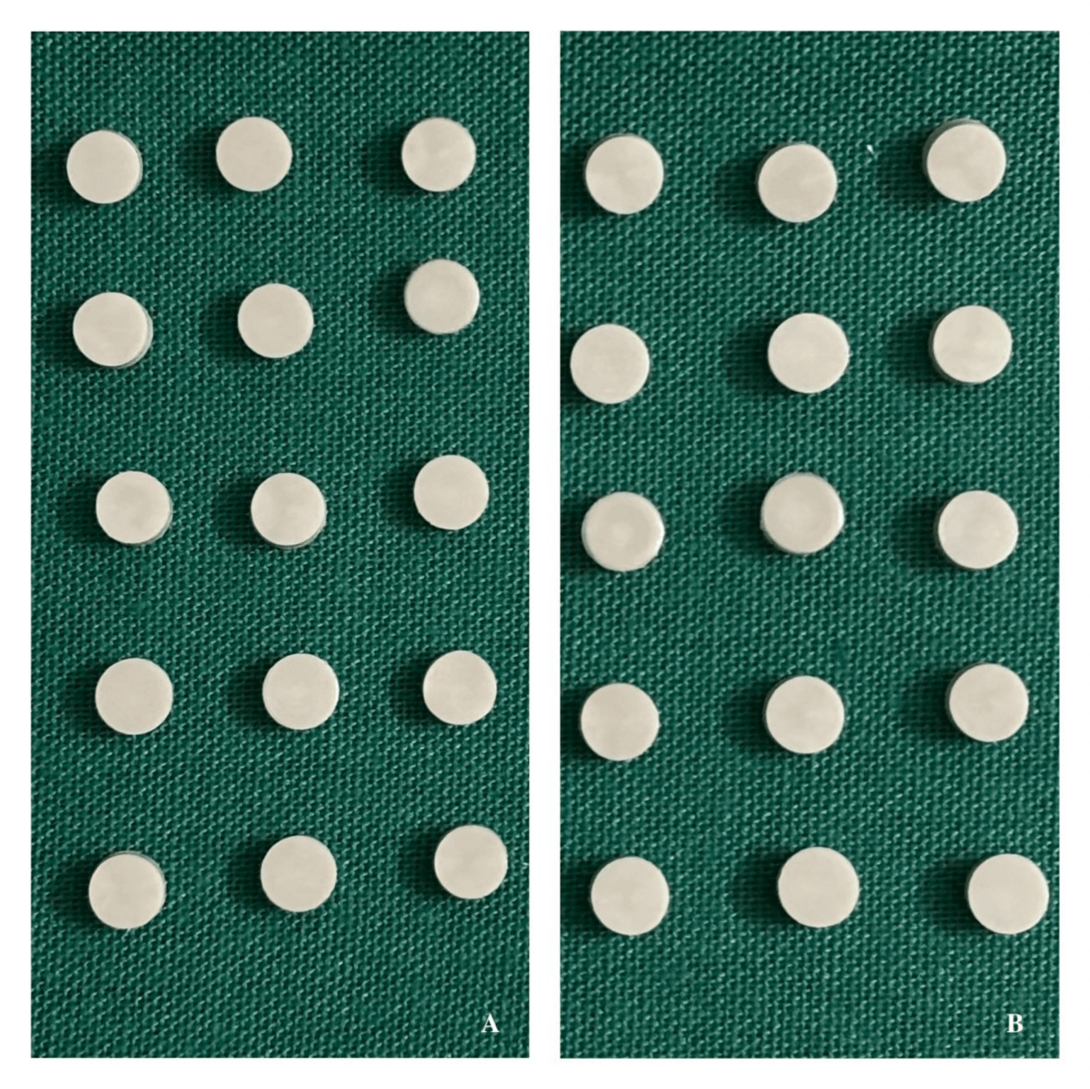
Unfilled and Filled Polyetheretherketone (PEEK) Samples A – Group A: Unfilled PEEK group, B – Group B: Filled PEEK group

This experimental in vitro study was conducted to evaluate and compare microbial colonisation on unfilled and glass-filled PEEK specimens before and after surface modification with plasma and laser treatments. Specimen fabrication was carried out using the injection moulding process at Srikrishna Polymers, Chennai, India. Surface treatments were performed at the Vellore Institute of Technology, Vellore, Tamil Nadu, India, and microbial analysis was undertaken at Genolites Private Limited, Coimbatore, Tamil Nadu, India, under controlled laboratory conditions. An a priori power analysis for one-way ANOVA (fixed effects, omnibus) was performed using G*Power (version 3.1). The study was based on an alpha error probability of 0.05, a statistical power of 0.90, and a large effect size (f = 0.80) in accordance with Cohen’s conventions for experimental studies, where substantial group differences are expected under controlled in vitro conditions [10]. With two primary groups (Group A: unfilled PEEK and Group B: 30% glass fibre-reinforced PEEK) (Figure 1), the required total sample size was calculated as 54 specimens (noncentrality parameter λ=2.98; critical F=2.98; actual power = 0.902). Each primary group (n = 27) was further divided into three treatment categories: untreated control, plasma-treated, and laser-treated. These were subsequently subdivided according to the bacterial strains tested (Escherichia coli, Streptococcus mutans, and Streptococcus oralis), resulting in nine subgroups per group (A1a-A3c and B1a-B3c). Each subgroup comprised three specimens (n = 3), totalling 54 specimens across 18 experimental subgroups.

Surface treatments

*Plasma Treatment*

**Figure 2:**
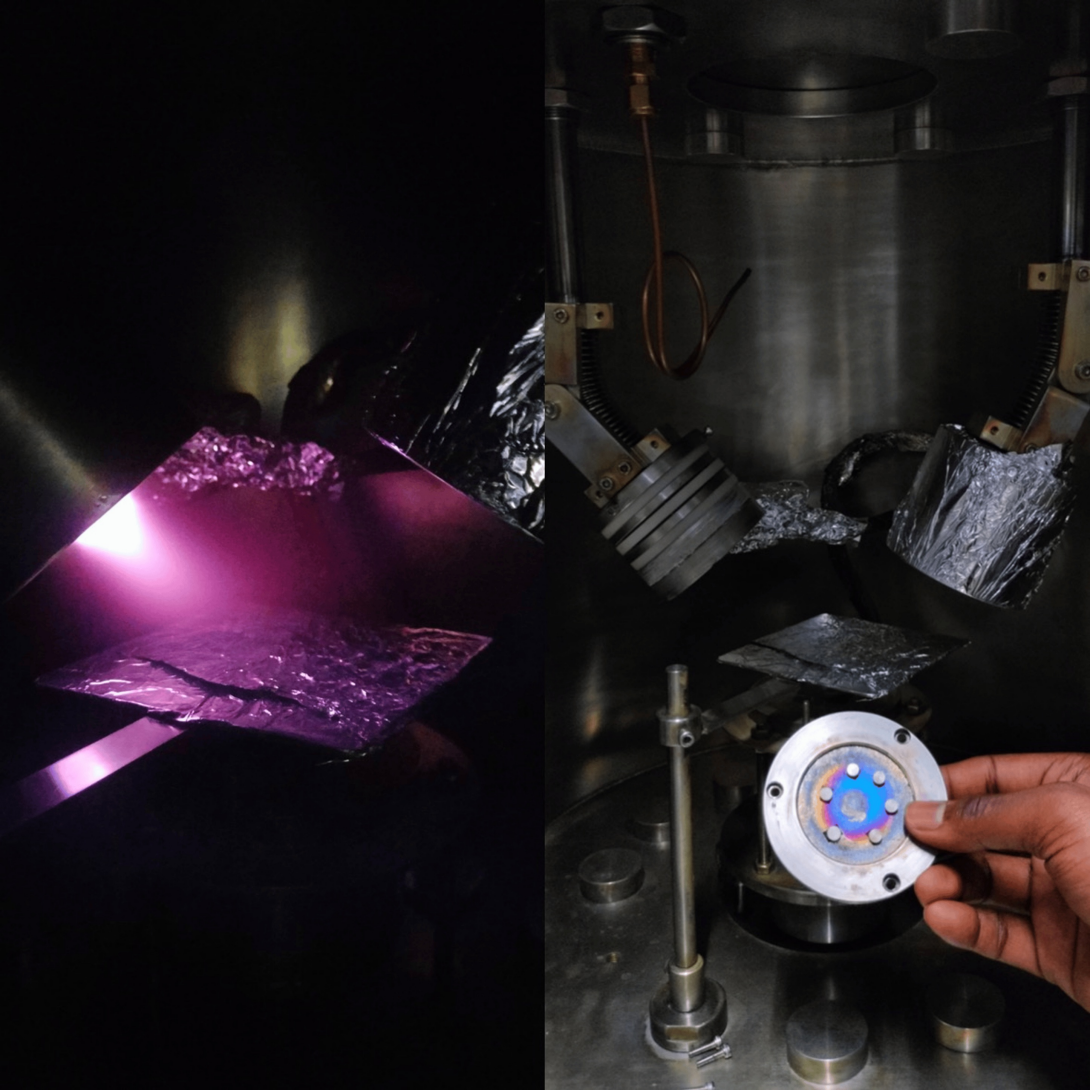
Plasma Surface Treatment (Argon)

Plasma modification of both unfilled and filled PEEK samples was carried out in a vacuum chamber evacuated to low pressure. Argon gas, chosen for its inert nature, was ionised to generate plasma, which physically altered the surface properties of PEEK without causing chemical interaction. Specimens, pre-cleaned with ethanol and distilled water, were exposed to the plasma for 30 seconds. Uniform exposure was ensured by positioning them centrally and evenly spaced within the chamber. Following treatment, the samples were allowed to return to ambient conditions (Figure 2).

*Laser Treatment*

**Figure 3:**
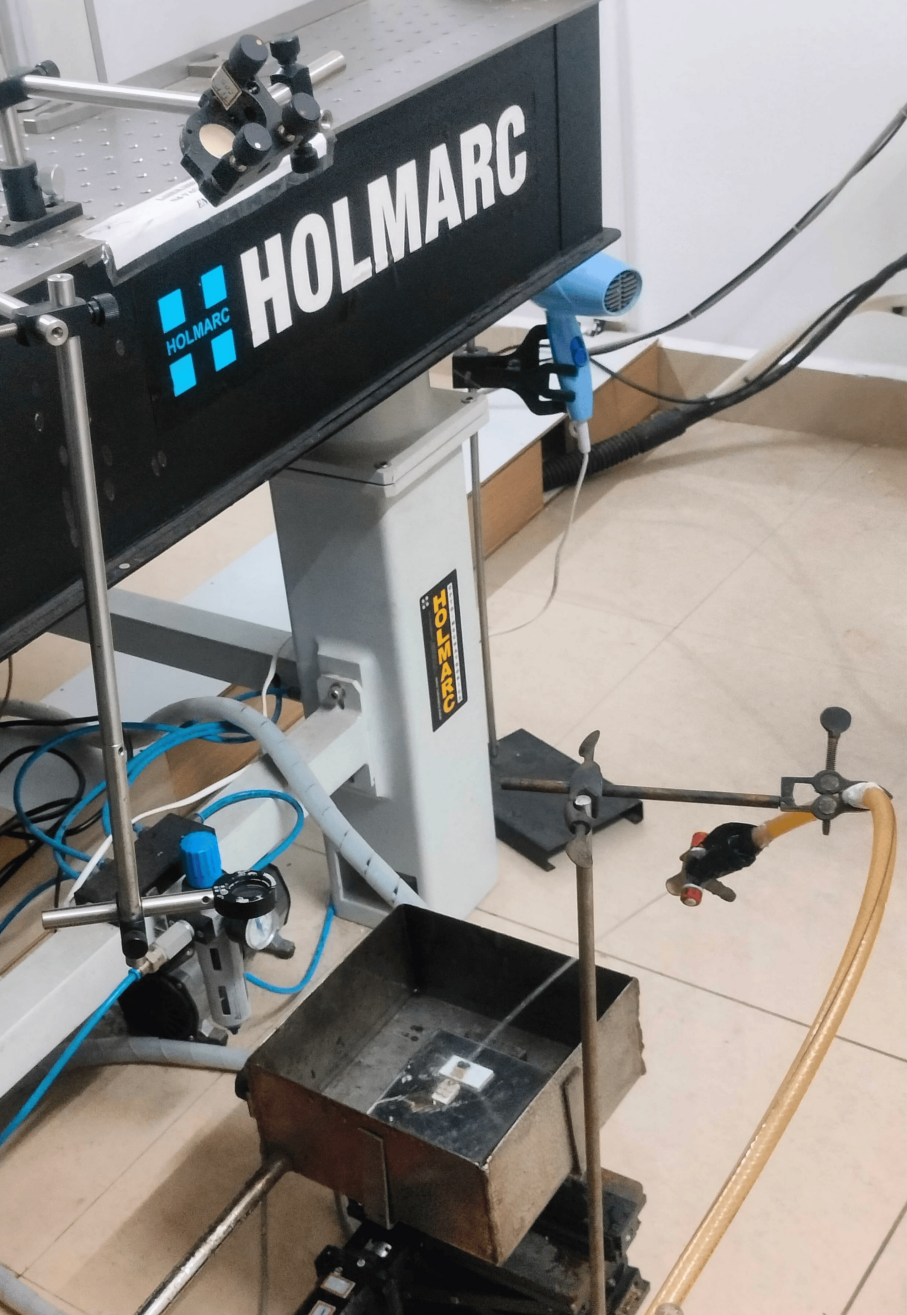
Laser Surface Treatment (Nd:YAG)

Laser surface modification was performed using a Q-switched Nd:YAG laser (Model BS EN 60825-1; Litron Lasers, Rugby, UK), selected for its precision and high-energy pulsed output. The laser operated at a frequency of 10 kHz with a pulse duration of 10 ns and a spot size of 1 cm². Specimens were irradiated in a non-contact mode, maintaining a focal distance of 50-200 mm to ensure uniform exposure across the surface. The applied fluence was approximately 0.20 J/cm² (within the working range of 0.10-0.30 J/cm²), sufficient to induce surface activation and moderate roughening without causing thermal degradation. Following irradiation, samples were allowed to cool naturally to room temperature to stabilize the material (Figure 3).

Surface characterization

**Figure 4:**
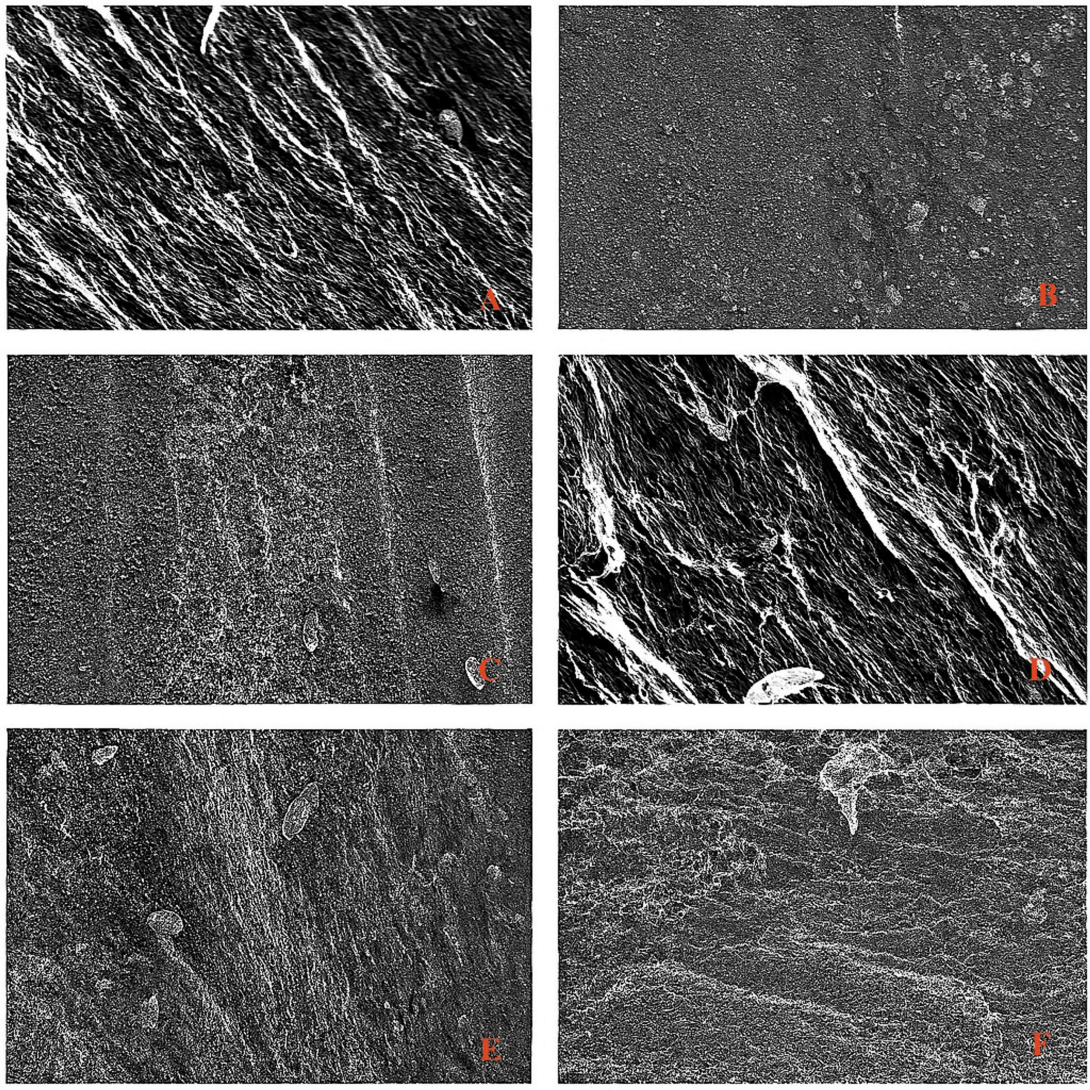
Scanning Electron Microscope (SEM) Images Showing the Surface Characterisation A – Unfilled PEEK B – Unfilled PEEK laser surface treated C – Unfilled PEEK plasma surface treated D – Filled PEEK E – Filled PEEK laser surface treated F – Filled PEEK plasma surface treated

Surface morphological analysis was conducted using a scanning electron microscope (SEM; Thermo Fisher Scientific Inc., Mumbai, India). Both treated and untreated samples were mounted on conductive stubs and imaged under standardised settings. Surface features such as roughness, porosity, and cracks were evaluated. ImageJ software (v1.53t) was used to quantify morphological parameters. For roughness evaluation, grayscale intensity profiles were generated, and 3D surface plots were created using a plugin. Thresholding allowed quantification of particle size, pore distribution, and crack geometry through the "Analyze Particles" tool (Figure 4).

Biofilm analysis

Biofilm formation on the surface of PEEK specimens was evaluated using a crystal violet (CV) biomass assay. Three clinically relevant microbial strains, Escherichia coli (MTCC 40), Streptococcus mutans (MTCC 890), and Streptococcus oralis (MTCC 2969), were selected as primary oral colonisers. These strains were obtained from the MTCC culture collection bank (Chandigarh, India) and preserved at -80 °C until use. For experimentation, each strain was revived in tryptone soy broth (TSB; HiMedia Laboratories Pvt. Ltd., Mumbai, India) and incubated overnight at 37 °C under anaerobic conditions. The overnight cultures were diluted 1:10 in fresh TSB and incubated for two hours in a shaking water bath at 37 °C to achieve the exponential growth phase. Sterilised PEEK discs were then placed into sterile 96-well polystyrene microtiter plates, and 200 µL of standardised bacterial suspension was added to each well. Plates were incubated at 37 °C for 24 hours under anaerobic conditions to allow biofilm development. Following incubation, non-adherent planktonic bacteria were carefully removed with a micropipette, and the specimens were washed three times with phosphate-buffered saline (PBS; HiMedia Laboratories) to remove loosely attached cells. Biofilms were fixed with 0.1% CV solution (HiMedia Laboratories) for one minute, after which excess stain was removed with PBS, and the samples were dried at 37 °C for two hours. Bound stain was subsequently solubilised with ethanol, and the eluates were transferred to fresh wells. Optical density (OD) was measured at 570 nm using a microplate reader (Synergy Hx Multi-Mode Microplate Reader, BioTek Instruments, Winooski, VT, USA). The OD values reflected the total biomass of biofilm on the PEEK surface, with higher values indicating greater microbial accumulation and lower values denoting reduced adhesion or enhanced antimicrobial activity of the treated specimens.

Live/dead cell determination

**Figure 5:**
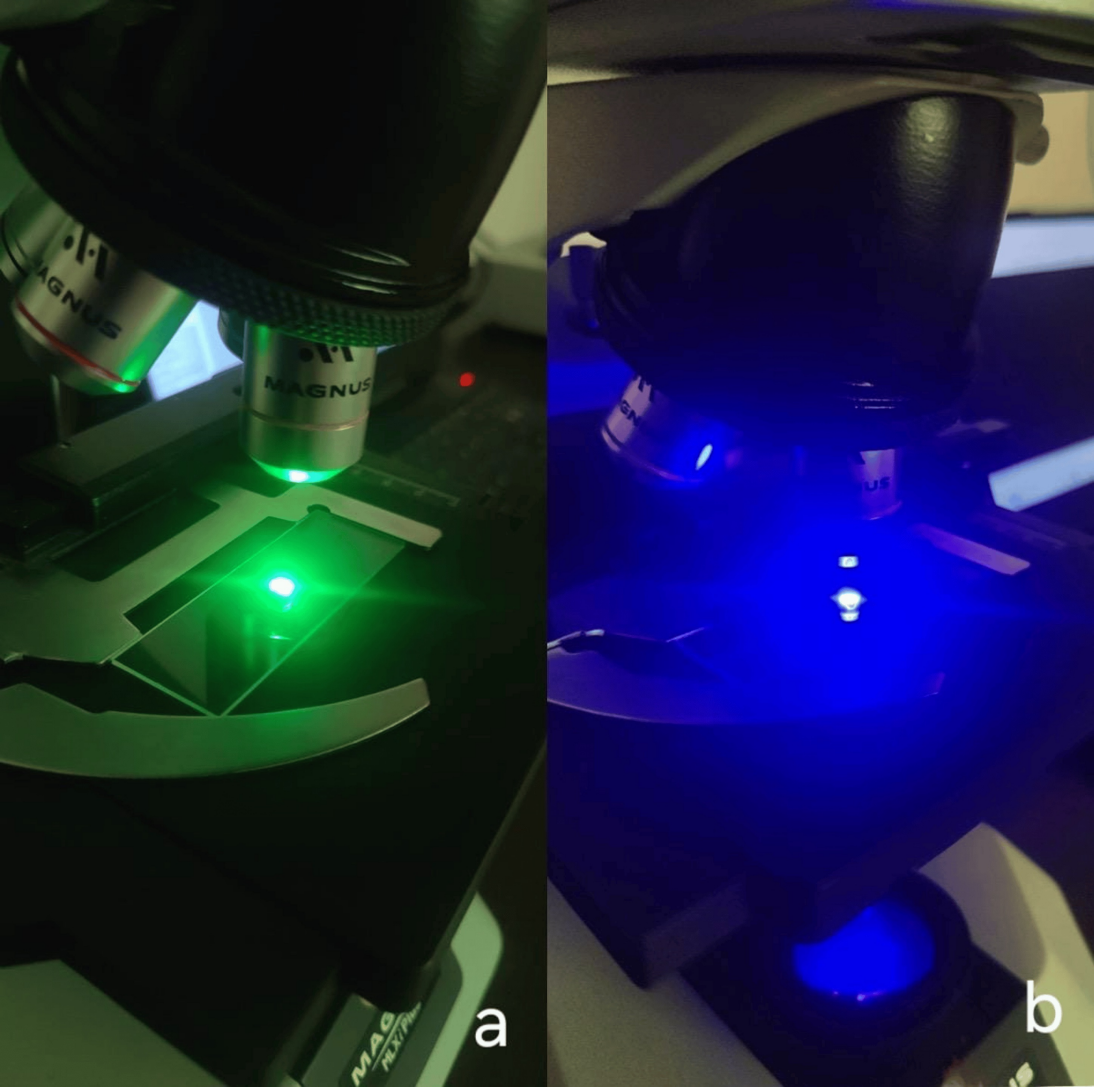
Viewing of Samples in MLXi Fluorescent Microscope a- Viewing of Samples at Green Light (580nm) b- Viewing of Samples at Blue Light (450nm)

Post-inoculation, microbial viability on PEEK surfaces was assessed using dual fluorescent staining with acridine orange (live cells) and ethidium bromide (dead cells). Samples were stained for 15 minutes and incubated for 24 hours, followed by PBS washes to remove excess stain. Specimens were mounted on slides, and imaging was performed under a fluorescent microscope (Magnus MLXiplus; Uvsar India Pvt. Ltd., Ghaziabad, India) using appropriate excitation filters (450 nm for green fluorescence and 580 nm for red fluorescence) (Figure 5). ImageJ was used to analyse fluorescence images by thresholding each channel and counting cell populations using the “Analyze Particles” function.

Statistical analysis

Data were analyzed using SPSS software (version 26.0; IBM Corp., Armonk, NY, USA). Normality of data distribution was assessed with the Shapiro-Wilk test. Biofilm biomass values (OD570 nm) were compared among groups using one-way analysis of variance (ANOVA). Live/dead fluorescence images were quantified in ImageJ (v1.53t) using channel thresholding and the “Analyze Particles” function to calculate the percentage of viable and non-viable cells; these results were presented descriptively as mean percentages. All data were expressed as mean ± standard deviation (SD), and statistical significance was set at p < 0.05.

## Results

Surface modification

**Figure 6:**
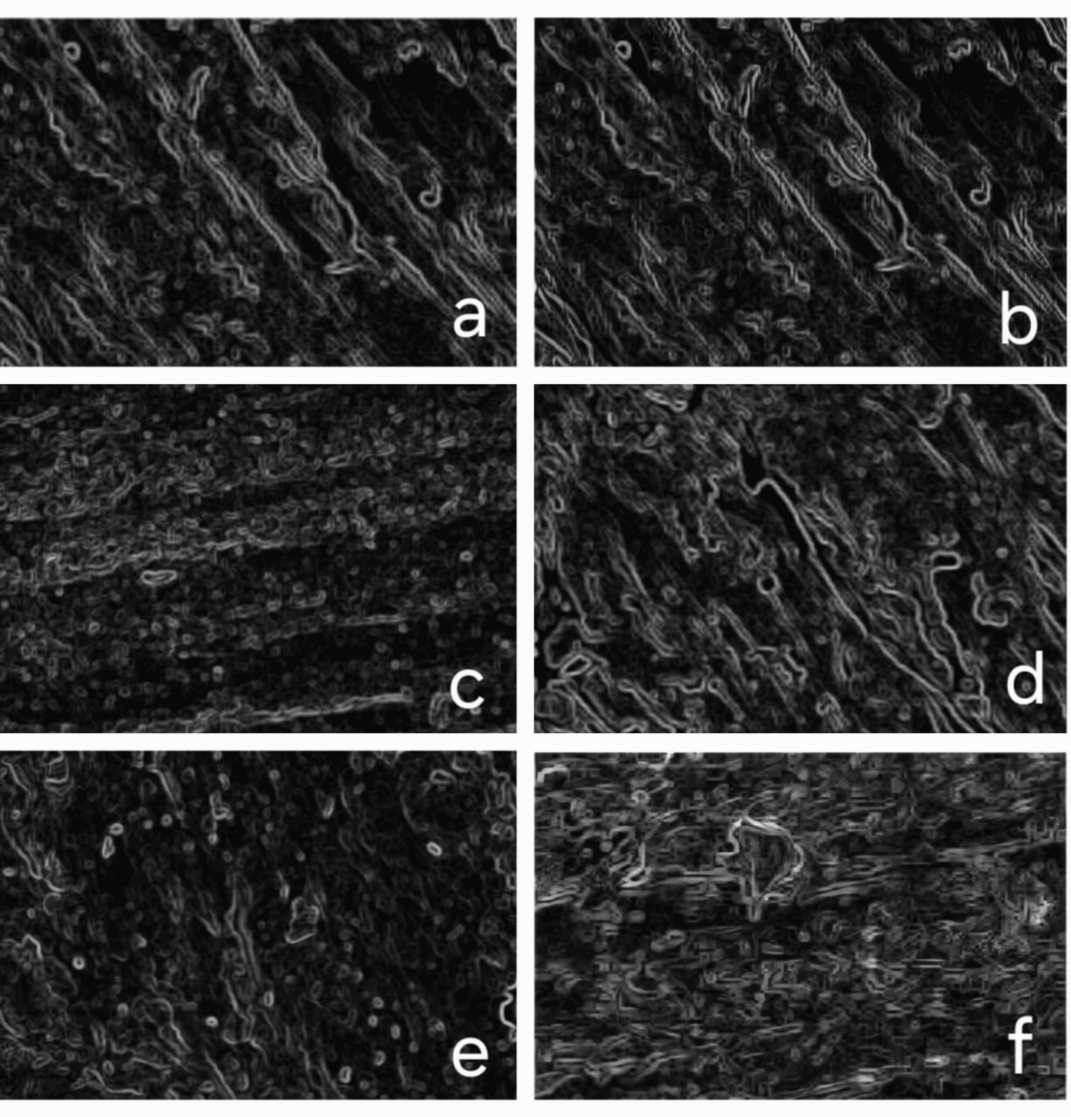
Edge Detection of Scanning Electron Microscope (SEM) Analysed Samples a- unfilled polyetheretherketone (PEEK) samples (control) b- unfilled laser surface-treated PEEK samples c- unfilled plasma surface-treated PEEK samples d- filled PEEK samples (control) e- filled laser surface-treated PEEK samples f- filled plasma surface-treated PEEK samples

Laser treatment effectively transformed irregular grooves into a uniform micro-structured pattern, particularly in unfilled PEEK due to the absence of reinforcing fillers (Figure 6). In filled PEEK, surface uniformity was less consistent owing to the heterogeneity introduced by filler particles. Plasma treatment produced a more uniform nanostructured surface with improved surface energy but lacked the pronounced micro-scale patterning seen with laser treatment. Edge detection and 3D reconstruction confirmed smoother, more refined topographies post-treatment, with unfilled PEEK showing greater surface consistency. Particle size analysis corroborated these findings, highlighting deeper and more defined features in laser-treated samples versus the finer textures from plasma treatment.

Biofilm formation

**Figure 7:**
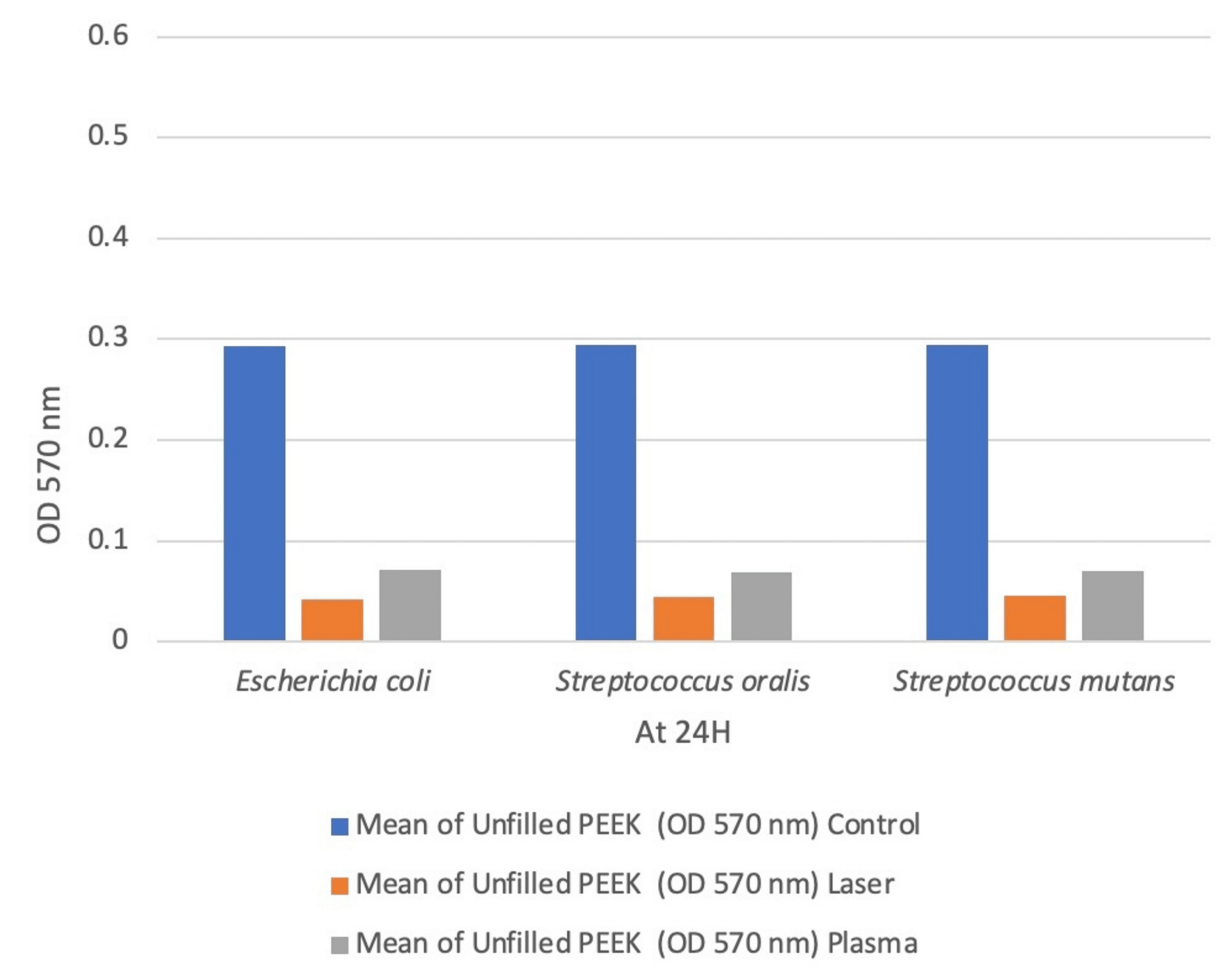
Graphical Representation of Mean Values for Biofilm Assay of Unfilled Polyetheretherketone (PEEK) (Group A)

**Figure 8:**
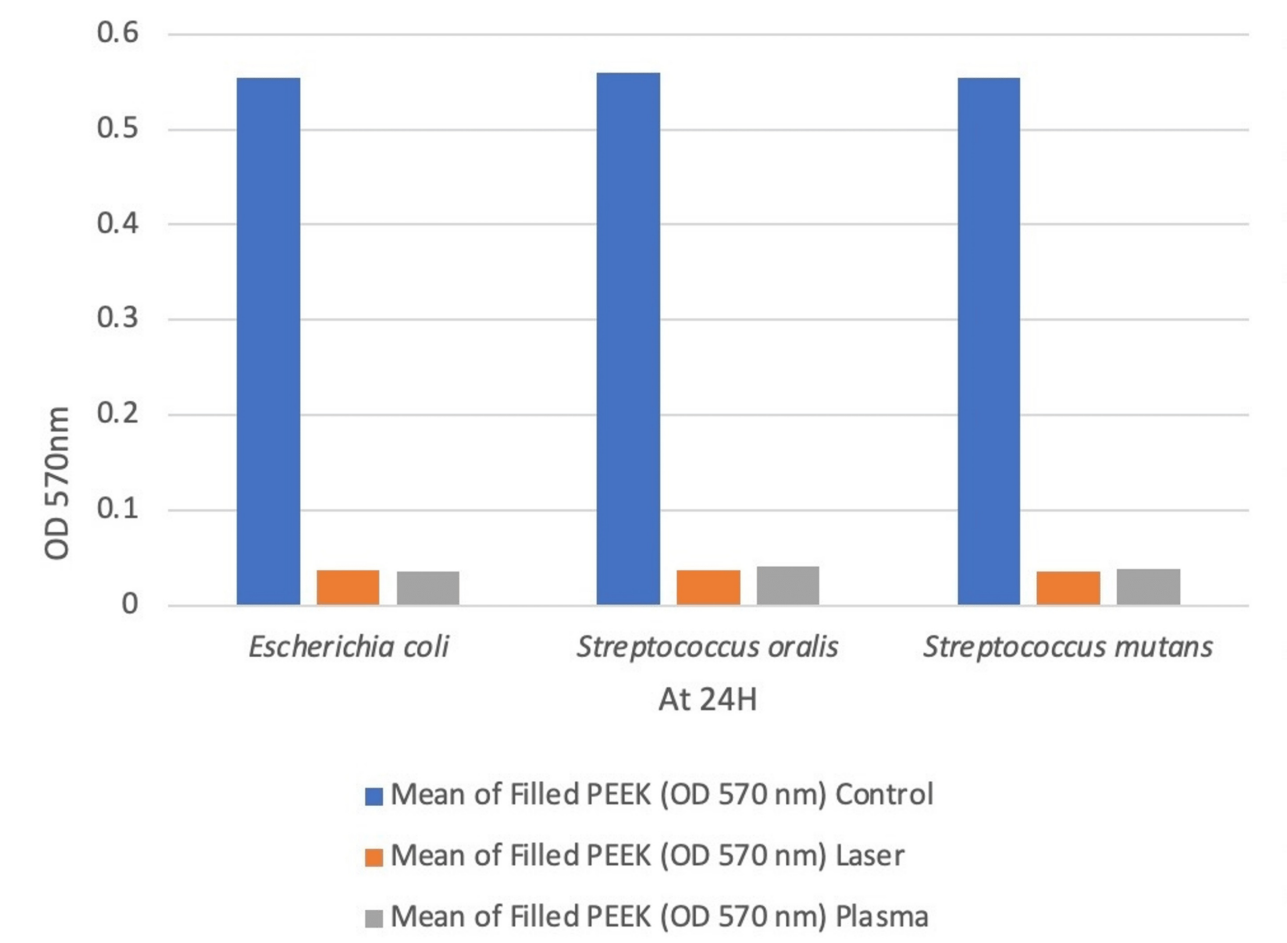
Graphical Representation of Mean Values for Biofilm Assay of Filled Group (Group B)

Biofilm biomass was quantified using OD570 nm readings, and statistical analysis was carried out using the one-way ANOVA test. Biofilm accumulation was highest in the untreated control across all bacterial strains (Table 1, Figures 7, 8). For Escherichia coli, the control group showed significantly higher biofilm than the laser (Δ = 0.517) and plasma (Δ = 0.518) groups (p < 0.001). No significant difference was observed between laser and plasma treatments (p = 0.975).In Streptococcus oralis, similar trends were observed, with significant reductions in both treatment groups (p < 0.001) and no difference between them (p = 0.919). For Streptococcus mutans, both treatments significantly reduced biofilm compared to the control (p < 0.001), with no significant intergroup difference (p = 0.815).

**Table 1:** Comparison of Mean Values for Biofilm Assay of Unfilled Polyetheretherketone (PEEK) (Group A) and Filled PEEK (Group B) Using One-Way ANOVA Test Values are expressed as mean OD570 readings. Statistical analysis was performed using one-way ANOVA. p < 0.05 was considered statistically significant. Group A: Unfilled PEEK; Group B: Filled PEEK.

Species	Mean of unfilled PEEK at OD 570nm (group A)			Mean of filled PEEK at OD 570nm (group B)			
	Control	Laser	Plasma	Control	Laser	Plasma	Statistics (P-Value)
E. coli	0.293	0.042	0.0708	0.554	0.0369	0.036	0.001
S. oralis	0.294	0.044	0.0683	0.563	0.0369	0.041	0.001
S. mutans	0.294	0.0455	0.0705	0.554	0.0362	0.039	0.001

Live/dead staining

**Figure 9:**
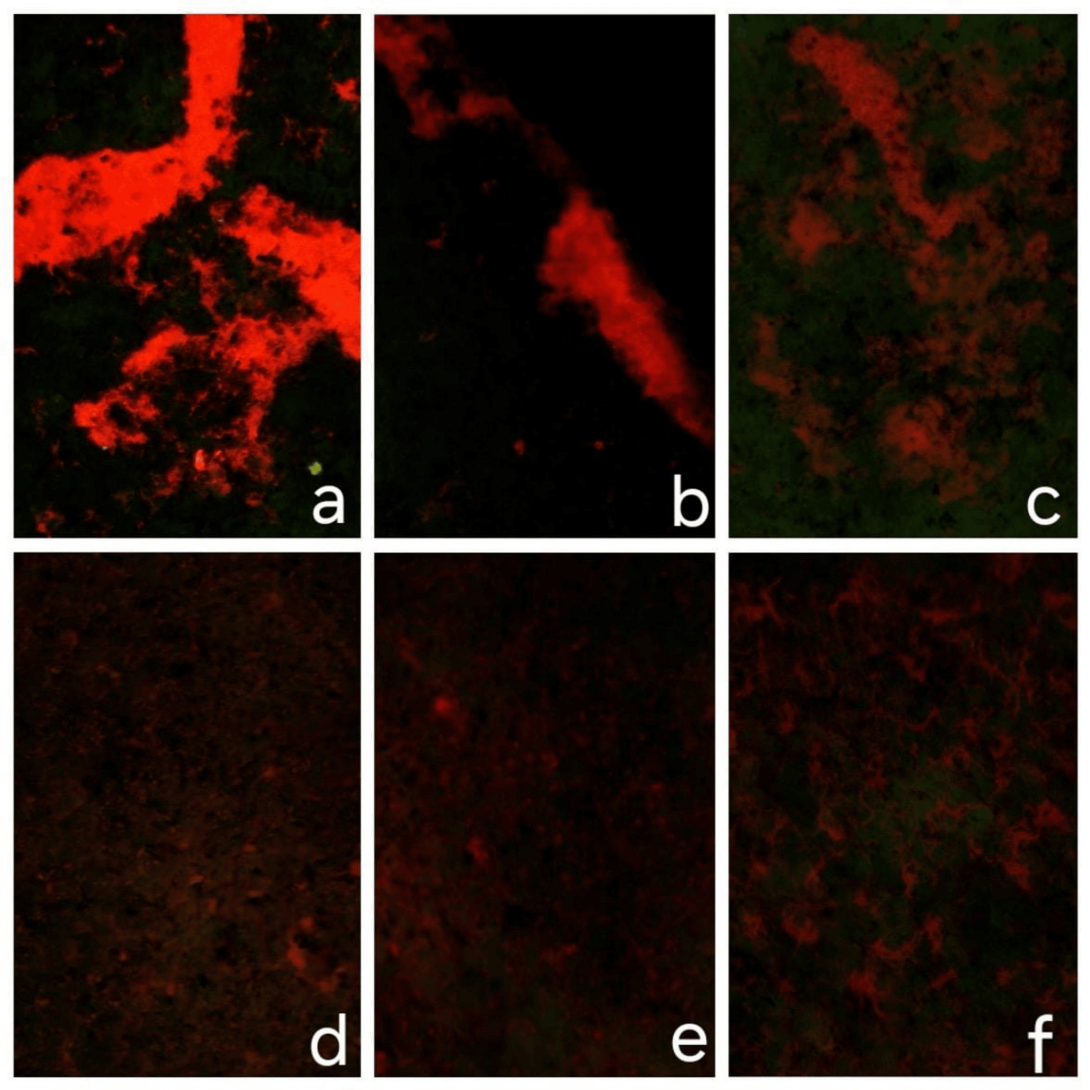
Live/Dead Staining of Samples a- unfilled polyetheretherketone (PEEK) sample (control) b- laser surface treated unfilled PEEK sample c- plasma surface treated unfilled PEEK sample d- filled PEEK sample (control) e- laser surface treated filled PEEK sample f- plasma surface treated filled PEEK sample

Surface treatments significantly reduced bacterial viability across all PEEK types and species (Figure 9). Escherichia coli showed the highest viability on untreated unfilled PEEK (80.12%), with a sharp decline after plasma (12.54%) and laser (11.90%) treatments. Filled PEEK further reduced viability (53.05%), with similar post-treatment reductions (plasma: 13.12%, laser: 12.82%). Streptococcus mutans viability dropped from 57.25% (unfilled) to 18.97% (plasma) and 11.85% (laser). Filled PEEK (48.15%) followed a similar trend, with further reductions after plasma (17.98%) and laser (12.09%). Streptococcus oralis exhibited the lowest viability on untreated unfilled PEEK (48.91%), which further declined after plasma (10.62%) and laser (8.17%). Filled PEEK (42.38%) also showed reduced viability, with additional decreases after plasma (18.37%) and laser (12.82%) treatments. Overall, both plasma and laser treatments consistently decreased bacterial viability across all PEEK groups and species.

## Discussion

This study was designed to evaluate the influence of laser and plasma surface treatments on the microbial adhesion and biofilm formation of unfilled and glass-filled PEEK. Surface topography and chemistry play a critical role in modulating microbial attachment on biomaterials, and the present results provide a detailed comparison of how two widely used surface modification techniques differentially impact bacterial behaviour on PEEK substrates. Scanning electron microscopy revealed that Nd:YAG laser treatment produced a consistent microstructured topography through localised melting and resolidification, while argon plasma treatment generated nanoscale surface texturing with relatively homogeneous distribution. These findings are similar to Akkan et al., where they compared plasma and short laser pulse treatment of medical-grade PEEK for controlled wetting, and they demonstrated that plasma treatment yields nanostructured surfaces conducive to wettability control, whereas short-pulse laser ablation results in microscale surface reorganisation [11]. Quantitative biofilm biomass assay and live/dead staining demonstrated that laser-treated surfaces exhibited significantly reduced microbial growth and viability across all three test organisms (Escherichia coli, Streptococcus mutans, Streptococcus oralis) compared to both untreated controls and plasma-treated groups. The antimicrobial efficacy of the laser-modified surfaces can be attributed to two synergistic effects: first, the induced microtopography physically disrupts bacterial colonisation by limiting initial attachment and subsequent biofilm maturation; and second, the introduction of oxygen-containing functional groups, such as hydroxyl and carboxyl moieties, enhances surface hydrophilicity and elevates surface free energy. This is similar to the findings of Wilson et al. [8], who reported that laser modification of PEEK leads to oxidation of carbonyl groups, contributing to a surface chemistry less favourable to bacterial adhesion. Additionally, our observation that Nd:YAG laser-treated PEEK exhibited greater microbial resistance than plasma-treated PEEK aligns with prior literature suggesting that microscale textures are less prone to biofilm development than nanostructures, which may offer increased contact points and topographical compatibility with bacterial cells.

The comparison between unfilled and filled PEEK also revealed a material-dependent effect on bacterial adhesion. Glass-filled PEEK consistently demonstrated lower biofilm formation and microbial viability than unfilled PEEK, regardless of surface treatment modality. This could be attributed to the antimicrobial properties associated with bioactive glass fibres, which are known to release calcium and silicon ions capable of altering the local microenvironment and disrupting bacterial homeostasis. Previous studies, including those by Jones et al. and Mu et al. [12,13], have shown that ion-releasing glasses, even in the absence of metallic dopants, can exert dose-dependent bacteriostatic effects. In the present study, the combined effect of glass reinforcement and surface treatment appeared to enhance the exposure of these fibres, potentially increasing ion exchange at the surface and contributing to the observed antimicrobial outcomes. The interaction between topographical modification and filler exposure appears to be synergistic in glass-filled specimens, further promoting bacterial inhibition. Live/dead fluorescence imaging provided additional insights into species-specific responses. Among all tested bacteria, E.coli exhibited the highest survival rate, particularly in untreated PEEK samples. As a motile Gram-negative species with a relatively thin peptidoglycan layer and efficient adhesion mechanisms, Escherichia coli demonstrates superior colonisation on hydrophobic or less structured surfaces. This is in agreement with the study by Etxeberria et al. [14], which indicated that Escherichia coli exhibits delayed yet sustained adhesion over time compared to Gram-positive organisms like Staphylococcus aureus. In our findings, however, surface treatment significantly suppressed this effect, underscoring the role of tailored surface engineering in mitigating even aggressive colonisers (Escherichia coli ), which was used in our study.

Taken together, the results of this study suggest that laser surface modification, particularly when applied to glass-filled PEEK, offers superior resistance to microbial colonisation. This enhanced resistance is likely attributed to a combination of induced microtopography, increased surface hydrophilicity, chemical functionalization, and the exposure of antimicrobial glass fillers. In contrast, plasma treatment, while effective in increasing wettability, showed relatively higher microbial adhesion, possibly due to nanoscale surface features offering favourable anchorage for bacterial cells. This observation aligns with findings by Cheng et al. and Wang et al. [15,16], who noted that nano-textured surfaces, although beneficial for eukaryotic cell adhesion, may also promote bacterial attachment depending on surface roughness and surface energy. These findings contribute to the growing body of evidence supporting surface engineering as a viable strategy to enhance the biological performance of high-performance polymers like PEEK, particularly in implant dentistry and prosthetic applications. Laser surface modification combined with glass reinforcement appears to be a promising approach for improving antimicrobial behaviour without compromising material integrity.

Despite these encouraging results, the study has certain limitations. Microbial colonisation was assessed under static in vitro conditions that do not fully replicate the dynamic oral environment, where salivary flow, masticatory forces, and immune responses play critical roles in biofilm development and material performance. The relatively short experimental timeframe limits conclusions regarding long-term efficacy or surface degradation over extended clinical use. Additionally, detailed surface chemical characterization was beyond the scope of this study, and the long-term stability of the functional groups introduced through laser or plasma treatment remains unclear. Future investigations using dynamic models, extended evaluation periods, and in vivo validation are necessary to better simulate clinical conditions and confirm long-term outcomes. It is also important to acknowledge that while surface modification enhances certain material properties, it can present drawbacks. These include potential inflammatory responses, gradual degradation or wear of surface coatings, increased complexity in manufacturing protocols, and possible incompatibility between the modified surface and the bulk material. A promising future direction involves incorporating bioactive plant-derived nanoparticles into the PEEK matrix. These nanoparticles can significantly improve material hardness and hydrophobicity, promoting favourable cell responses and osteoblastic differentiation during osseointegration [17]. Exploring such biofunctional composites, particularly in combination with surface treatments, could lead to the development of next-generation PEEK-based implants with enhanced mechanical and biological performance.

Clinical implications

Surface modifications of PEEK using laser or plasma can reduce biofilm formation and enhance adhesion, improving the performance of denture bases and dental implants. Nd:YAG laser treatment of PEEK denture bases may limit microbial colonization and increase prosthesis longevity. Plasma-treated implants benefit from enhanced osseointegration, and combining with antibacterial coatings can further reduce bacterial adhesion. Glass-filled PEEK shows reduced microbial growth, making it a promising material for prosthetic and implant applications where infection control is critical.

## Conclusions

This study highlights the effectiveness of laser and plasma treatments in modifying PEEK surfaces to enhance antimicrobial properties. Laser treatment, particularly with an Nd:YAG laser, was the most effective in reducing bacterial viability and biofilm formation due to microstructural modifications that disrupt bacterial adhesion. Plasma treatment also improved antimicrobial performance, primarily through surface activation and wettability changes. Additionally, glass-filled PEEK exhibited enhanced bacterial resistance, likely due to the antimicrobial effects of embedded glass fibers. These findings support the application of laser-treated PEEK in biomedical fields where antimicrobial properties are crucial, while plasma treatment remains a viable option for surface functionalization and tissue integration.
